# Impact of the Coronavirus Disease 2019 (COVID-19) Pandemic on Medical Interpreters: A Qualitative Focus Group Study

**DOI:** 10.1177/24731242251407900

**Published:** 2026-02-15

**Authors:** Marin P. Melloy, Faiza Aziz, Chloe Botsford, Sherri Fong, Lauren McPherson, Kathryn Freeman

**Affiliations:** 1 University of Minnesota Medical School, Minneapolis, Minnesota, USA.; 2 Ohio University Heritage College of Osteopathic Medicine, Athens, Ohio, USA.; 3 Department of Family Medicine and Community Health, University of Minnesota Medical School, Minneapolis, Minnesota, USA.

**Keywords:** interpreter, telemedicine, non-English language preference (NELP), limited English proficiency (LEP), COVID-19, team-based care

## Abstract

**Introduction:**

The coronavirus disease 2019 (COVID-19) pandemic changed the health care experience, impacting medical interpreters and the non-English language preference (NELP) patients they serve. NELP patients experienced increased isolation, barriers to care, and worse health outcomes. Interpreters faced rapid changes in care provision, including high patient volumes, increased illness severity, and transition to virtual and remote care. We aim to explore the experience, challenges, and recommendations of medical interpreters providing care during the COVID-19 pandemic.

**Methods:**

Five virtual focus groups, with a total of 24 professional medical interpreters, were conducted using a semi-structured guide. Transcripts were then coded for thematic analysis with an iterative debriefing process to refine codes and themes.

**Results:**

The challenges faced by participants during the COVID-19 pandemic included a rapid transition to remote interpretation, personal and patient safety concerns, elevated mental and emotional burden, and limited institutional support. Despite these challenges, participants felt immense pride in their role and the care provided. They describe limited opportunities for connection and support and a lack of professional recognition and detailed opportunities to further support interpreters in the future.

**Conclusion:**

Interpreters identified challenges in adapting to virtual and remote work, ensuring safety and quality of care, and maintaining mental health.

**Health equity implications:**

Interpreters suggest developing further health care worker training, highlighting best practices for NELP and interpreted visits, supporting interpreter professional development, and increasing interpreter visibility and recognition as next steps to improve care quality. Further insight into the perspective of NELP patients is needed to support high-quality, equitable patient care.

## Background

The onset of the coronavirus disease 2019 (COVID-19) pandemic changed how patients, clinicians, and communities experience health care in the United States, presenting unique challenges for medical interpreters and the non-English language preference (NELP) patients they serve. The pandemic exposed racial and socioeconomic inequities, including higher hospitalization rates for NELP patients or those who prefer a non-English language for their care.^1–3^ During the COVID-19 pandemic, NELP patients experienced higher rates of intensive care unit admission, intubation, and in-hospital death compared with English language preference (ELP) patients, including those of the same cultural background.^4^ Medical interpreters were at the forefront of these experiences, whether interpreting for NELP patients in person or virtually.^5,6^

Many human aspects of care became increasingly challenging during the COVID-19 pandemic. Physicians, nurses, public health specialists, and other health care workers (HCWs) described high levels of fatigue, stress, and burnout during the pandemic.^7–9^ The contributing factors included uncertainty in medical knowledge surrounding the virus, increased patient volume and illness severity, longer work hours, and unsafe staffing levels, which affected care quality.^8–15^

Professional medical interpreters were essential for communication between NELP patients, families, and clinical teams during the COVID-19 pandemic. Research on interpreter experience during the pandemic is limited but notes barriers in interpreters’ ability to effectively work remotely and subsequent decreases in care quality.^5,6,16,17^ There is a paucity of research encompassing the experience of interpreters supporting NELP patients during the COVID-19 pandemic. We aim to describe the perspective of professional medical interpreters caring for NELP patients, identifying challenges and strengths while highlighting opportunities for future improvement.

## Methods

### Recruitment and sampling

Professional medical interpreters working in Minnesota, USA, were recruited via an electronic recruitment flyer sent to organizations and clinicians who regularly interact with medical interpreters via local health care systems, interpreter agencies, and other professional organizations. Interested participants were directed to an electronic screener, and responses were collected and managed using REDCap electronic data capture tools hosted at the University of Minnesota.^18,19^ The eligibility criteria included professional medical interpreters with >3 years work experience, who were employed more than 20 hours per week, and with experience interpreting both virtually and in person. Recruitment was intentionally broad to capture participants from a variety of languages, cultural backgrounds, geographic locations, and clinical settings. This study was deemed Not Human Research by the University of Minnesota’s Institutional Review Board.

### Participants

A total of 24 medical interpreters participated in five focus groups. The demographics collected included age, sex, languages interpreted, type of employer, and community size (Table 1).

**Table 1.** Participant Characteristics

Characteristics	Count (%)
Age	
18–20	7 (29.2)
21–40	9 (37.5)
41–60	7 (29.2)
Decline to share	1 (4.2)
Sex	
Female	14 (58.3)
Male	9 (37.5)
Decline to share	1 (4.2)
Languages interpreted (select all that apply)	
Spanish	12
Russian	3
Arabic	2
Chinese/Mandarin	2
American Sign Language	1
Bulgarian	1
Hmong-White	1
Japanese	1
Nepali	1
Portuguese	1
Taiwanese	1
Ukrainian	1
Type of employer (select all that apply)	
Self-employed/independent contractor	15
Local interpreter organization	6
National interpreter organization	2
Single clinic/hospital	1
Health care system	11
County or state employed organization	0
Other	3
Community size^a^ (select all that apply)	
Small town (<5,000 people)	1
Large town (5,000–25,000 people)	3
Suburban (25,000–100,000 people)	10
Urban (major city > 100,000 people)	18

a Community size refers to the population of the community where the interpretation was happening.

Participants (n = 24) included professional medical interpreters participating in virtual focus group sessions. Participants were directed to select all that applied in the following categories: language interpreted, type of employer, and community size. Some participants interpreted for multiple languages (e.g., Spanish and Portuguese), worked for multiple employers (e.g., for a health care system but also as an independent contractor for some interpreting assignments), and worked in communities of multiple sizes (e.g., both suburban and urban areas). For this reason, the sums of these categories do not add up to 24.

### Data collection

Virtual focus groups were held between March and June 2023, with sessions recorded and transcribed via Zoom (Version 5.14.0). Participants provided written and verbal consent before each session. Sessions were 90–120 minutes long and conducted using a semi-structured guide led by two authors. Participants responded to prompts verbally and using the chat function. Questions focused on participants’ views of the interpreter role, virtual and in-person modalities, and experience during the COVID-19 pandemic. Following each session, participants completed a demographic survey and received a $50 gift card.

### Analysis

Focus group transcripts were coded for thematic analysis using an inductive approach based on grounded theory coding.^20^ The initial coding structure was developed during interim analyses after the completion of the first three focus groups. Once codes were developed, a minimum of three separate authors coded each transcript using NVivo 14.^21^ We used an iterative process of consensus building via paired debriefing, followed by a large group discussion to refine codes from each transcript based on common agreement. Coding summaries were developed for each thematic category, written by one author and reviewed by a second to ensure the inclusion of key concepts. Focus groups were conducted until the point of data saturation.

## Results

Participants described their experiences interpreting at the height of the COVID-19 pandemic in a variety of ways. When asked to give one word encapsulating the feeling of being an interpreter during the pandemic, the most common adjectives expressed were “challenging,” “stressful,” and “isolated” (Fig. 1).

**FIG. 1.**
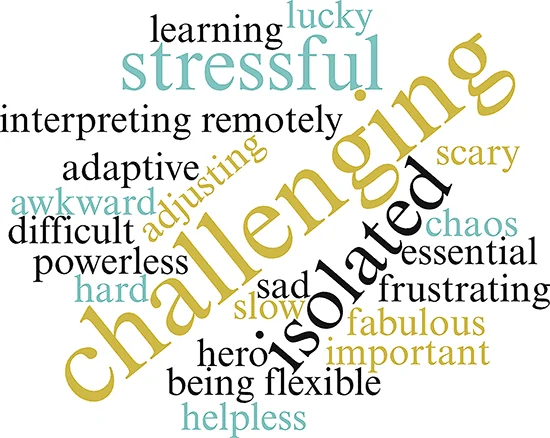
Word map reflecting words chosen by participants (n = 24) to describe interpreting during the COVID-19 pandemic. The word size is based on the frequency, with large words being mentioned more frequently. The most frequently mentioned words were challenging (n = 5), isolated (n = 2), and stressful (n = 2).

Several themes arose surrounding the challenges faced by interpreters during the COVID-19 pandemic (Table 2). These themes included the challenges of remote interpretation, safety concerns, mental and emotional burden, and lack of support. Although the challenges may have been preexisting, their impact was amplified by the pandemic. Despite these challenges, participants declared a strong sense of purpose and pride in their pivotal work supporting NELP patients. They maintained the value of high-quality medical interpretation and identified opportunities for growth and advocacy to enhance patient care, support interpreters, and uplift the profession.

**Table 2.** Themes Identified by Professional Medical Interpreters around the Experience of Interpreting during the COVID-19 Pandemic along with Illustrative Quotes Not Included in the Text

Theme	Illustrative quotes
Challenges of remote interpretation	“So many patients that didn’t have the possibility of or didn’t know even how to get to Zoom, how to use a platform. This came as such a surprise […] Some of us had to learn very fast. And I don’t think that the patients had the same opportunity or the ability, or the possibility or the means to learn how to do this so fast. So I think that it was stressful, because you know that your patient needs you, and they cannot get in, they cannot participate.”-Focus group 2, Participant ID 34
	“There’s like the mental burden of working over the phone, too, where you’re maybe trying to hear. And you’re spending so much energy trying to hear them that it kind of takes away from the energy that you’re able to spend, you know, actual [*sic*] doing your interpreting job.”-Focus group 3, Participant ID 5
	**Compared with before the COVID-19 pandemic:** “When pandemic [*sic*] started, we had to switch to these different modes of interpreting, to different virtual platforms. It was a challenging, not for ourselves, but also for the providers, and also for the clients or the patient, they are not able to navigate the platform. A lot of times we had to jump in to help them to educate and then to navigate the system. So getting services was challenging.”-Focus group 2, Participant ID 28
Safety concerns	“We need to be far apart. How can we do that in a room that is so tiny? […] It was just so hard for the patient too, through the mask. It was hard […] for them to hear us and for us to hear them, and at the same time we could not be close enough to the patients to talk to them.”-Focus group 2, Participant ID 34
	**Compared with before the COVID-19 pandemic:** “We were not prepared. And then, all of a sudden, throw all those all those PPE requirements. Now you show up as an in person with PPE and a lot of […] the clients that have limited English proficiency are sometimes very soft spoken, or sometimes they like to express themselves with gestures. So how do you explain all that if you can’t see?”-Focus group 1, Participant ID 43
Mental and emotional burden	“Mentally, it’s still very draining. I don’t know how everybody else feels about it, but I take things home. There’s family members that you know can’t be there, we have to extubate, we have to, you know. Just call it for what it is [*sic*], and it’s very hard because we have to tell someone [to] express to us how they feel, and they’re saying their last [words].”-Focus group 2, Participant ID 16
	**Compared with before the COVID-19 pandemic:** “And as you know, we did that before [*sic*] Covid. But for some reason now it’s been more often, and it’s been, you know children, adults, elderly you name it. I mean it has happened, and I feel like in that way, we’re a little bit more prepare [*sic*].”-Focus group 2, Participant ID 16
Lack of support	“Well, I could share that in my case, the environment [...] in regards to how we work completely changed. We went from a hundred percent in person interpreting to actually get furloughed, which baffled me because the patient population still needed us. But they had no, the tools were not really set up to protect us, nor to provide the services that we needed to provide. So […] it was a huge struggle.”-Focus group 5, Participant ID 59
	**Compared with before the COVID-19 pandemic:** “What made it difficult for me during Covid was like medical stuff […] like the receptionist, they made it difficult for interpreters understanding our role. We have a role. We follow a guideline just like in any medical professional walking through the door […] Sometimes they did not provide PPE for us, knowing that, you know there’s a patient with Covid. So it that’s when I meant by chaos, because they made it difficult for the interpreters.”-Focus group 2, Participant ID 33
Sense of value and pride	“I just felt really very fortunate to be able to continue working and to be able to provide such a needed service, and to be able to continue to support my community and support my coworkers in that role.”-Focus group 4, Participant ID 64
	**Compared with before the COVID-19 pandemic:** “I think there were some adjustments with that, for example, especially as at the beginning of the pandemic, and there were so many changes with how clinics were handling things. For example, if a patient was ill, or if they were going in for like a routine check, if there was a specific door they needed to go into, or a different entrance they needed to go into, and I found myself shifting my role a lot to try to inform those patients of what these policies were both […] so the providers had less on their plates that they were already dealing with so many variables themselves, and also to try to protect, […] patients. I didn’t want the language barrier for patients to be the issue for them to go into the wrong door and expose people who might be vulnerable, or [*sic*] that sort of thing, so I [*sic*] know that I kind of took the role of trying to explain some of those procedural adjustments that we’re going on to clients.”-Focus group 2, Participant ID 51

### Theme 1: Challenges of remote interpretation

With the rapid, unanticipated onset of the COVID-19 pandemic, many health care systems were caught off guard, without plans for the continuation of interpreter services. Quickly, remote or virtual interpretation via telephone or video became commonplace; yet, many participants received little or no formal training in the new technology, format, or communication platforms. Novel interpretation modalities forced interpreters to learn in real time without guidance, as health care systems, clinicians, nurses, and patients simultaneously adapted. Participants voiced frustrations with the lack of sufficient information and technology training.

“We were untested at that point. We didn’t have processes in place for how to work with this new dynamic […] There was active communication and conversation about how to do this work, how to do it effectively. But nobody had the specific strategy or answer.”-Focus group 5, Participant ID 60

They noted a loss of communication cues and nonverbal input critical to their work as interpreters with the change to virtual formats.

“Technology is important. But it cannot take the place of human beings and communication […] Behavior is so important. How people talk, the gestures, the things that they express without words. That is something that so far, technology hasn’t been able to provide us in the same way.”-Focus group 4, Participant ID 62

Participants watched patients similarly struggle to utilize the new technology to access care. Many participants attributed the stress surrounding remote interpretation during the COVID-19 pandemic to losing control of multiple aspects of their jobs and lives. Similar to other adults working remotely, some participants struggled to balance work and family responsibilities, including online schooling for their children. Participants expressed work-related stressors, such as burnout, mental drain, and technology fatigue, similar to other HCWs. Participants reported that these stressors harmed their ability to perform well in their role.

“We are the person in the room that has to be calm and collected. But this was stressful in my particular experience, because of any and every aspect that was not under my control. Practitioners were not pausing for us.”-Focus group 2, Participant ID 34

### Theme 2: Safety concerns

Participants voiced major safety concerns during the pandemic regarding both their physical safety and the safety of interpretation quality. Concerning physical safety, participants feared becoming sick with COVID-19 or infecting their loved ones. Space limitations and a shortage of personal protective equipment (PPE) exacerbated these fears. Remote options felt safer yet at times inadequate, like when patients needed breathing support, and an in-person interpreter was required to hear them over the noise of medical equipment.

“At the back of your mind, you are like, I have to stay as healthy as possible for the patients, so I can help them, but also for my family and for myself […] and that’s the fear.”-Focus group 1, Participant ID 43

Interpreters noted disruption in the visit flow and pacing. Time constraints on remote visits or harried interactions prevented appropriate pauses to ensure complete understanding of medical information. When in person, participants reported that masks and PPE blocked facial expressions, limited lip reading, and muffled voices. When working over the phone, participants noted a loss of nonverbal cues and challenges with background noise, including difficulty identifying the speaker and inserting themselves appropriately when needed.

“But it’s really hard to see if the people are understanding what I said. The way I interpret it, was it right? Was it the right word? Did it really click, or didn’t it? I don’t know. They gasp, and it could be a good thing or a bad thing, I don’t know. But if I see a face, that really makes a difference”-Focus group 1, Participant ID 23

### Theme 3: Mental and emotional burden

Participants shared that interpreting during the COVID-19 pandemic came at a great mental and emotional cost with vicarious trauma. For participants who shared cultural backgrounds with patients, there was a heightened sense of responsibility, as the pandemic disproportionately affected underrepresented populations, including NELP communities. They described the major impacts of COVID-19 visitor restrictions on patients, family members, and interpreters. Interpreters often assumed roles traditionally supported by in-person family members, such as providing emotional support and explaining situations to ill and disoriented patients. This role was particularly important for NELP patients with low health literacy or from collective cultures with heavy familial interdependence.

“It’s not just interpreting for the patient, it’s also interpreting for their family members, and I think that that was a challenge, and that made it very helpless where their loved one was the main person that was, quarantined or isolated in this area, and then they didn’t have that natural support system.”-Focus group 3, Participant ID 14

### Theme 4: Lack of support

Participants described feeling a general disconnect from the health care team. They watched as public attention was directed toward physicians and nurses, overlooking the contributions of interpreters and other HCWs also providing essential care. Despite the integral role of interpreters in equitable health care for NELP patients, many health care systems failed to protect interpreters from COVID-19, whether physically with protective gear or financially with job stability. Some participants were furloughed despite a constant stream of patients who needed their legally required services.

“So very, very stressful, very draining, and we felt at times that the health systems were not supporting enough the [*sic*] interpreters. […] Everybody focused on doctors and nurses. But little was spoken about or said about interpreters, our job […] the challenges that the interpreter or the interpreters faced during these times, and the stressors […] because we needed to be there. Wearing all this, you know, the face mask and the N95. At least in my area, it was absolutely devastating.”-Focus group 5 Participant ID 72

## Thematic Overlap

Four central themes were deeply connected by feelings of uncertainty, fear, and responsibility. Physical safety concerns led to the accelerated adoption of remote interpretation despite a lack of organizational support and inadequate training. Participants saw a disregard for patient and provider preferences in the interpreter format, which led to questionable interpretation quality, with an increased risk of misunderstanding and safety concerns for patients. This, along with witnessing patient deaths, family tragedies, and community losses, contributed to the mental and emotional burden for interpreters. Participants voiced uncertainty about future job security and fears that experienced interpreters may leave the profession due to a lack of organizational support, underappreciation of their expertise, and overreliance on technology.

### Theme 5: Sense of value and pride

Despite these challenges, participants described a strong sense of purpose in supporting NELP patients and remain hopeful for the future of medical interpretation. They viewed providing interpretation as a professional responsibility that allowed patients to be heard and communicate in times of illness. During the COVID-19 pandemic, participants remained actively working and were able to assist patients in accessing care broadly during the pandemic, from routine services to psychiatric care, emergency, dental, laboratory testing, and hospital services. They provided virtual and in-person care during high-risk situations, ensuring that communication pathways remained available. Participants expressed that supporting critically ill patients in their community, while very challenging, was meaningful.

### Recommendations

Participants shared opportunities for improvement going forward. First, they urged for the continued improvement in technology and its adaptation to support consistent access to interpreter services in both virtual and in-person formats. They emphasized increased HCW training as an opportunity to improve the interpretation flow and experience, focusing on best practices for working with interpreters, such as attention to volume and pacing, and improved understanding of interpreter roles. They stressed the value of integrating interpreter and patient perspectives into quality improvement processes to create workflows that better serve patients.

Conversations repeatedly led back to the need for further interpreter support, including resources for connection and well-being. Multiple participants described hearing their own experiences echoed by others during the focus groups as therapeutic and expressed a desire for additional support structures to share information, provide professional development, and facilitate advocacy efforts. Participants stressed the importance of institutional support for these endeavors and advocated for appropriate interpreter recognition and wages in an effort to recruit and retain interpreters in the field.

“The only way to bring professionals to this particular field is to treat interpreters like professionals. And therefore, there are many things that that institutions could do, like invest resources on making sure that there is professional development for those who are bilingual staff, who could potentially become interpreters, as well as compensating interpreters properly, and having an environment where interpreters feel supported and as part of the medical team.”-Focus group 5, Participant ID 59

## Discussion

Previous studies have focused on the challenges the COVID-19 pandemic brought for other frontline HCWs,^7^ but the perspective of professional medical interpreters is lacking. Clinicians, nurses, and interpreters all faced challenges adapting to new technologies and remote care modalities, maintaining safety, managing high patient volumes, and dealing with stress and burnout.^8–14^ Yet, for interpreters, structural support was lacking, including education on new developments, policies, and protocols; support for well-being; and recognition of their dedication and sacrifice. They felt significant professional responsibility for their patients and pride in their ability to provide high quality and essential skills, but limited access to information, agency in the process, and opportunities for feedback contributed to fatigue, frustration, and burnout.

The provision of virtual medical care increased during the COVID-19 pandemic, and telephone and video interpretations were integral for communication with NELP patients. Despite this, systemic barriers, including long wait times for virtual interpreters, inflexible scheduling systems, and technical issues, caused difficulties for both patients and medical staff.^22,23^ NELP patients faced the added challenge of connecting with both a provider and an interpreter, often using English-only virtual platforms,^3^ and experienced disparities in health care access due to the limited availability of electronic devices, internet, and overall digital skills needed to access telemedicine.^24–26^

As virtual interpretation remains commonplace in the wake of the COVID-19 pandemic, there is a continued need for timely technology access and training for interpreters to successfully perform their role. Patients would also benefit from additional technology resources and support. Professional organizations, public health departments, insurance providers, and health care organizations could be key partners to provide these services. Incorporating interpreters in virtual care design and implementation can improve access and functionality for patients and families.

Similar to many HCWs providing care during the COVID-19 pandemic, interpreters felt the competing demands of personal safety and professional duty to provide the highest quality care. Initially, interpreters had limited access to protective equipment, and later, they experienced prolonged exposures to critically ill patients. Various studies attributed increased levels of fear and anxiety across HCWs to multiple factors, including increased personal and familial risks of infection.^8,10,12–14,27^ Participants expressed heightened safety concerns stemming from limited knowledge about how to stay safe, indicating a need for information-sharing tools that include interpreters and other frontline employees who contract with health care organizations.

Despite different roles, medical interpreters and bedside nurses similarly described the emotional burden of providing individual patient care during the COVID-19 pandemic.^8,15^ Both communicated regularly with and invested in individual patients, including palliative and end-of-life care cases, with increased rates of emotional fatigue and exhaustion.^26,28^ HCWs from diverse backgrounds described an elevated awareness of the link between ethnicity and severe illness/death.^8,14^ This, alongside experiences of racism and systemic inequity, may disproportionately affect interpreters, who often come from and care for patients from similar ethnic and cultural backgrounds. Further research on end-of-life and critical illness conversations for diverse individuals, with improved support for families, nurses, and interpreters, will support the continued challenges of aging populations beyond the COVID-19 pandemic.

Multiple participants spoke of the challenge of witnessing firsthand the impact of the virus on their communities. Opportunities for additional interpreter support can be drawn from research on other HCWs, which identified protective factors and mitigators of harm during the COVID-19 pandemic.^8,15^ These include facilitating connections with colleagues, opportunities for interdisciplinary collaboration, recognition of accomplishments and sacrifices, frequent education, and strong leadership.^8^

Overall, interpreters felt immense pride in the hard work and care they provided during the COVID-19 pandemic. They also, at times, felt unrecognized and underappreciated despite their critical role. In each group, interpreters expressed a desire for the recognition of the vital nature of their work, as well as their personal efforts and sacrifices during the pandemic. They see the value of the unique interpreter experience, skills, and knowledge, which could improve policies, protocols, and systems that affect NELP patients. Further resources, education, professional respect, and integration within the health care system beyond the COVID-19 pandemic have the potential to build the interpreter profession, creating both a higher-quality and more sustainable working environment for interpreters and an improved NELP patient experience and outcomes going forward.

### Strengths and limitations

The study strengths include the diversity of the research team’s backgrounds in medicine, public health, and interpretation. This study is one of few to focus on the interpreter perspective and included a diverse group of interpreters in regards to language, location, practice, and employer, providing a wide range of viewpoints. However, both the number of participants and focus groups were small—one included only three participants. While our participants interpreted in 12 different non-English languages, the majority of participants were Spanish language interpreters (representative of the largest NELP population in Minnesota, USA).^29^ Our research team members also primarily practice within large academic health centers with a physician-heavy perspective, with minimal community, private clinic, or rural experience, limiting the generalizability of results. Multiple participants and physicians worked together previously, impacting the power differential and increasing the risk of bias.

### Health equity implications

Going forward, interpreters see additional connection, professional education, and appropriate interpreter recognition as ways to build the profession. They urge for the continued adaptation of technology and systemic workflows with interpreter and patient input to improve medical interpretation experiences.

While the COVID-19 pandemic brought awareness to the existing patterns and challenges in interpreter work and created new ones, many of these findings can be used to inform and improve practice and policies in the post-COVID era. Goals include the following:

- Ensuring a workplace culture that is welcoming for interpreters, allowing for increased connection, recognition, professionalism and better integration into the health care team^30^
- Providing and promoting accessible opportunities for high-quality training and ongoing professional education for both interpreters and professionals who work with interpreters to improve staffing, access, and partnership^30,31^
- Investing in high-quality, reliable, easy-to-use technology to facilitate telehealth and remote interpretation, including for patients^30^
- As COVID emergency funding disappears and pressures for cost savings increase, maintaining or improving reimbursement for interpreter services and developing sustainable protocols and policies to ensure continued access, especially to in-person interpreters, remain critical for high-quality, equitable care patient care

## Authors’ Contributions

F.A., L.M., and K.F. were involved in initial idea generation, literature review, and project development. M.M., F.A., S.F., L.M., and K.F. contributed to the study design, focus guide development, and participant recruitment. C.B. and K.F. served as focus group facilitators, and M.M., F.A., S.F., and L.M. acted as secondary facilitators. S.F. and K.F. developed initial analysis codes, and transcript coding and theme development and review involved all authors. F.A. completed the initial summary of COVID-19-related codes. M.M. developed the article outline, Method sections, and led the writing process, including major contributions to the literature search, thematic development, and the Introduction, Results, and Discussion sections. K.F. also contributed substantially to the literature search, Introduction, and Discussion sections. All authors participated in draft writing, editing, and revisions. All authors have read and approved the final article.
